# Identification and Interpretation of eQTL and eGenes for Hodgkin Lymphoma Susceptibility

**DOI:** 10.3390/genes14061142

**Published:** 2023-05-24

**Authors:** Yeeun An, Chaeyoung Lee

**Affiliations:** Department of Bioinformatics and Life Science, Soongsil University, 369 Sangdo-ro, Dongjak-gu, Seoul 06978, Republic of Korea

**Keywords:** expression quantitative trait loci, gene expression, mixed model, Hodgkin lymphoma, Reed–Sternberg cell

## Abstract

Genome-wide association studies (GWAS) have revealed approximately 100 genomic signals associated with Hodgkin lymphoma (HL); however, their target genes and underlying mechanisms causing HL susceptibility remain unclear. In this study, transcriptome-wide analysis of expression quantitative trait loci (eQTL) was conducted to identify target genes associated with HL GWAS signals. A mixed model, which explains polygenic regulatory effects by the genomic covariance among individuals, was implemented to discover expression genes (eGenes) using genotype data from 462 European/African individuals. Overall, 80 eGenes were identified to be associated with 20 HL GWAS signals. Enrichment analysis identified apoptosis, immune responses, and cytoskeletal processes as functions of these eGenes. The eGene of rs27524 encodes ERAP1 that can cleave peptides attached to human leukocyte antigen in immune responses; its minor allele may help Reed–Sternberg cells to escape the immune response. The eGene of rs7745098 encodes ALDH8A1 that can oxidize the precursor of acetyl-CoA for the production of ATP; its minor allele may increase oxidization activity to evade apoptosis of pre-apoptotic germinal center B cells. Thus, these minor alleles may be genetic risk factors for HL susceptibility. Experimental studies on genetic risk factors are needed to elucidate the underlying mechanisms of HL susceptibility and improve the accuracy of precision oncology.

## 1. Introduction

Hodgkin lymphoma (HL), distinguished from non-Hodgkin lymphoma (non-HL) by the presence of Reed–Sternberg (RS) cells, is one of the most common lymphomas. Research efforts have been made to discover the corresponding risk factors for susceptibility to HL, such as age [1], sex [2], family history [3], viral infection (Epstein–Barr virus [4], and human immunodeficiency virus [5]), autoimmune disease [6], pollution exposure [7], and cigarette smoking [8]. In particular, genetic factors have been greatly concerned because of the large concordance within the family. For example, a cohort study for 54 years with 57,475 first-degree relatives of 13,922 HL patients in Denmark, Finland, Iceland, Norway, and Sweden showed that a risk estimate of HL in first-degree relatives of a patient was 3.3-fold greater (95% confidence interval = 2.8–3.9) than that in the general population [3]. A case–control study with 128 childhood HL cases and 848 controls in France reported a significant association of HL with its family history (odds ratio = 5.4; 95% confidence interval = 1.3–22.0) [9]. Nevertheless, knowledge regarding genetic risk factors is limited, especially when compared with the extensive understanding of other cancers, including non-HL.

Early studies have demonstrated that the RS cells in patients with HL may have crippling mutations in genes encoding immunoglobulin (Ig) [10,11]. These studies revealed that RS cells originate from pre-apoptotic germinal center B cells that evade apoptosis. Differential gene expression was reported between HL cases and controls, including in genes involved in the evasion of apoptosis via constitutive signaling activity of nuclear factor kappa B (NF-κB; *NFKBIA* [12,13], *NFKBIE* [14], *TNFAIP3* [15,16], *REL* [17,18], *MAP3K14* [19], and *BCL3* [20]), JAK/STAT (*JACK2* [21], *SOCS1* [22], *STAT6* [23], and *PTPN1* [24]), and PI3K/AKT (*ITPKB* [25] and *GNAI13* [25]), alongside immune response escape by the downregulation of human leukocyte antigen (HLA; *PD*-*L1* [26], *PD*-*L2* [26], *B2M* [27], and *CIITA* [28]). A key complication of such gene expression studies is the low levels of available RS cells (0.1–10%) originating from the tumor microenvironment [29]. Over the last decade, an improvement in high-throughput sequencing technology has led to genome-wide association studies (GWAS) elucidating >100 quantitative trait loci (QTL) associated with susceptibility to HL across the human genome [30,31,32,33,34,35,36,37,38,39]. Nevertheless, the corresponding mechanisms underlying susceptibility to HL remain unknown.

The objectives of this study are to identify expression genes (eGenes) associated with the HL GWAS signals reported in previous studies and investigate the regulatory functions of expression QTL (eQTL) on eGenes using omics approaches.

## 2. Materials and Methods

### 2.1. Gene Expression and Genotype Data

eQTL were identified using gene expression and genotype data from 373 Europeans (CEU, Utah Residents with Northern and Western European ancestry; GBR, British; FIN, Finnish; TSI, Tuscans in Italy) collected by the 1000 Genomes Project Consortium (E-GEUV-3) [40]. Gene expression levels were measured in reads per kilobase of transcript per million mapped reads (RPKM) of mRNA in lymphoblastoid cell lines (LCLs) using the Illumina HiSeq2000 platform by the GEUVADIS consortium [41]. Gene expression data were normalized by probabilistic estimation of expression residuals in a Bayesian framework [42,43]. All gene expression data available for 22,721 genes were used to identify the eGenes. We retrieved the corresponding genotypes obtained from the 1000 Genome Project, in which whole genome sequencing, targeted exome sequencing, and microarray chip sequencing data were integrated [44]. The current eQTL analysis employed the exclusion criteria of minor allele frequency <0.05 and Hardy–Weinberg disequilibrium (*p* < 10^−6^) for nucleotide sequence variants.

### 2.2. GWAS Signals for HL

HL GWAS signals were obtained from the National Human Genome Research Institute–European Bioinformatics Institute GWAS Catalog (https://www.ebi.ac.uk/gwas/, accessed on 16 July 2021) [45]. To examine eQTL for HL-specific lymphoma, 580 SNPs were retrieved for both HL (76; EFO_0000183) and non-HL (504; EFO_0005952). The non-HL included follicular lymphoma, diffuse large B-cell lymphoma, extranodal nasal NK/T cell lymphoma, central nervous system lymphoma, and marginal zone B-cell lymphoma. Among these, only independent signals with a minor allele frequency >0.05 and Hardy–Weinberg equilibrium (*p* > 10^−6^) were selected. After excluding common signals for both the HL and non-HL groups, we conducted a transcriptome-wide eQTL analysis with HL-specific signals.

### 2.3. Statistical Analysis for Identifying eQTL

Transcriptome-wide association analysis was conducted to identify eGenes associated with previously reported HL GWAS signals. The association between each eQTL and eGene was statistically tested in a mixed model framework that explains the polygenic effect, thus reducing spurious eGenes [46]. This polygenic effect is explained by the genomic covariance structure of individuals and can be included as a random effect in the corresponding analytical model [47]. The mixed model was produced under the assumption of an additive genetic model, as follows:

(1) y=xβ+g+ϵ

where y is the n × 1 vector with elements of standardized gene expression levels, n is the number of individuals with gene expression levels, β is the scalar of the fixed candidate nucleotide variant effect, x is the n × 1 design vector for the fixed effect, g is the n × 1 vector with random polygenic effects, and ε is the n × 1 vector with random residual elements. The design vector x consists of 0, 1, and 2 according to the number of minor alleles in the corresponding individual. The random variables in the mixed model were assumed to have normal distributions, with the following parameters:

(2) $$y\sim N\left(0,G\sigma_{g}^{2}+I\sigma_{\varepsilon }^{2}\right)$$

(3) $$g\sim N\left(0,G\sigma_{g}^{2}\right)$$

(4) $$\varepsilon \sim N\left(0,\;I\sigma_{\varepsilon }^{2}\right)$$

where G is the n × n genomic similarity matrix, $\sigma_{g}^{2}$ is the polygenic variance component, I is the n × n identity matrix, and $\sigma_{\varepsilon }^{2}$ is the residual variance component. Elements ($g_{jk}$) of the genomic similarity matrix G consist of all pairwise genomic similarity coefficients [47] and were calculated using genotypic information of individuals, as follows:

(5) $$g_{jk}=\frac{1}{n_{v}}\overset{n_{v}}{\underset{i=1}{\sum }}\frac{\left(\tau_{ij}-2f_{i}\right)\left(\tau_{ik}-2f_{i}\right)}{2f_{i}\left(1-f_{i}\right)}$$

where $g_{jk}$ is the genomic similarity coefficient between individuals j and k, $n_{v}$ is the number of all variants that contribute to the genomic similarity, $\tau_{ij}$ and $\tau_{ik}$ are the numbers of minor alleles at nucleotide variant i of individuals j and k, respectively, and $f_{i}$ is the frequency of the minor allele [48]. The fixed nucleotide variant effect can be underestimated through a partial redundancy in random polygenic effects via nucleotide variants in linkage to the candidate variant. To avoid this problem in the current eQTL analysis, the genomic similarity coefficient was estimated by excluding all nucleotide variants located on the same chromosome as the candidate variant.

Polygenic and residual variance components were estimated using restricted maximum likelihood (REML). The average information algorithm was used to obtain REML estimates of the variance components. Subsequently, the fixed candidate nucleotide variant effect was obtained with variance component estimates by solving Henderson’s mixed model equations [48]. The fixed nucleotide variant effect was tested for eQTL. All processes were replicated for each potential eGene–eQTL pair. Mixed model analysis was conducted using GCTA software at https://gump.qimr.edu.au/gcta (accessed on 16 July 2021) [49].

Each eGene–eQTL association was determined by multiple testing with a significance threshold value of 4 × 10^−5^, which was slightly more conservative than the Bonferroni correction. Linkage disequilibrium (LD) blocks were constructed using Haploview [50], with the algorithm established by Gabriel et al. [51], to determine the significantly associated variant as an independent eQTL signal.

### 2.4. Functional Analysis of eGenes

For the established eGenes from eQTL analysis, we examined their corresponding biotypes using Ensembl GRCh37 (https://grch37.ensembl.org/index.html (accessed on 17 July 2021)) [52]. Their functions with categories annotated in Swiss-Prot (UniProtKB; https://www.uniprot.org (accessed on 26 July 2021)) [53] and Gene Ontology (GO; http://geneontology.org (accessed on 26 July 2021)) [54]. To discover potential latent pathological mechanisms of HL, gene enrichment analysis was conducted with these eGenes using Enrichr (https://maayanlab.cloud/Enrichr (accessed on 6 August 2022)) [55] in the Python programs from GSEApy (version 0.10.8.) [56]. The enrichment analysis used 7821 GO terms in the gene set libraries of biological processes (6036 terms), cellular components (511 terms), and molecular functions (1274 terms) [54]. The significance of eGene enrichment was determined using the threshold value that was adjusted for multiple testing.

### 2.5. Functional Analysis of eQTL

The regulatory functions of eQTL in eGene expression were predicted using deep learning-based artificial intelligence methods. We utilized the ExPecto program to predict tissue-specific transcriptional effects of sequence variants within the LD blocks of cis-eQTL. Transcriptional functions were predicted by the convolutional neural network model trained with all 2002 available profile data containing histone marks, transcription factors, and DNA accessibility information (https://github.com/FunctionLab/ExPecto (accessed on 20 October 2022)) [57].

The roles of the identified functional variants were also predicted using the Sei programs (https://github.com/FunctionLab/sei-framework (accessed on 20 October 2022)) [58]. These programs used 21,907 peak calls of transcription factor binding (9471), histone marks (10,064), and chromatin accessibility (2372); this information was taken from the international consortia of the Cristrom Project [59], ENCODE [60], and Roadmap Epigenomics [61]. A convolutional neural network was employed to predict specific regulatory functions. To specify regulators binding to functional variants, atSNP (http://atsnp.biostat.wisc.edu/search (accessed on 3 January 2023)) was used [62]. This tool predicts specific transcription factor binding to functional variants via statistical testing of the maximum binding affinity score using subsequences encompassing the variant position within a position weight matrix that is preliminarily assigned from the ENCODE [60] and JASPAR [63] motif libraries. Differential binding affinity by allele was determined using the likelihood ratio test.

## 3. Results

### 3.1. eQTL Analysis

**Figure 1:**
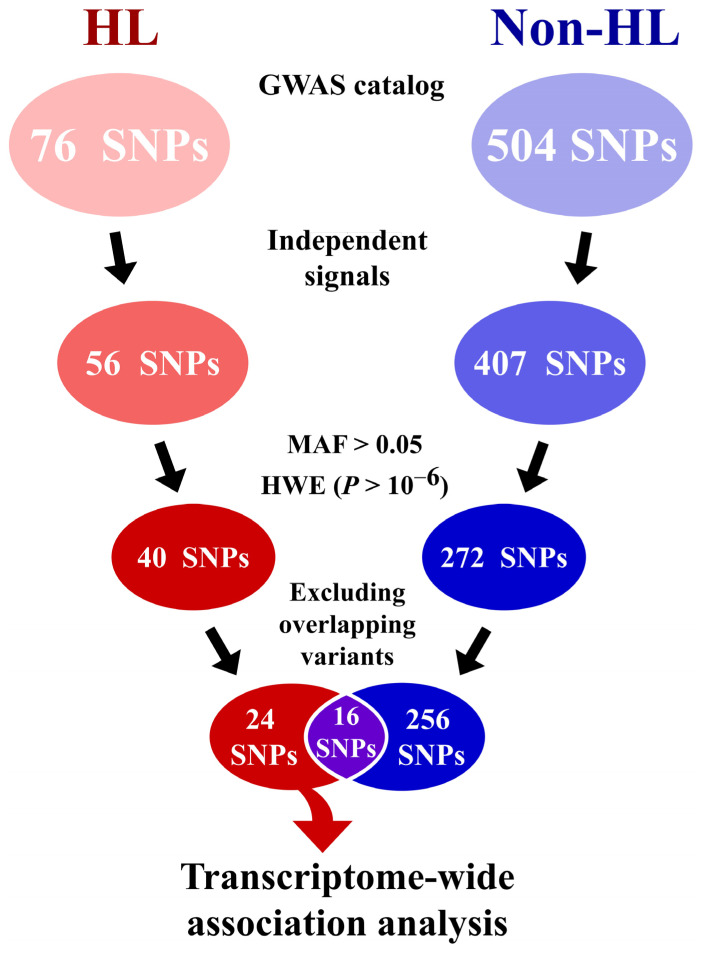
Flow diagram illustrating the data extracting procedure of Hodgkin lymphoma (HL) genome-wide association study (GWAS) signals that were analyzed in the current transcriptome-wide expression quantitative trait loci (eQTL) study. Data for HL, non-Hodgkin lymphoma (non-HL), and both groups are presented in red, blue, and purple, respectively. Abbreviations: SNP, single nucleotide polymorphism; MAF, Minor allele frequency; HWE, Hardy–Weinberg equilibrium.

**Figure 2:**
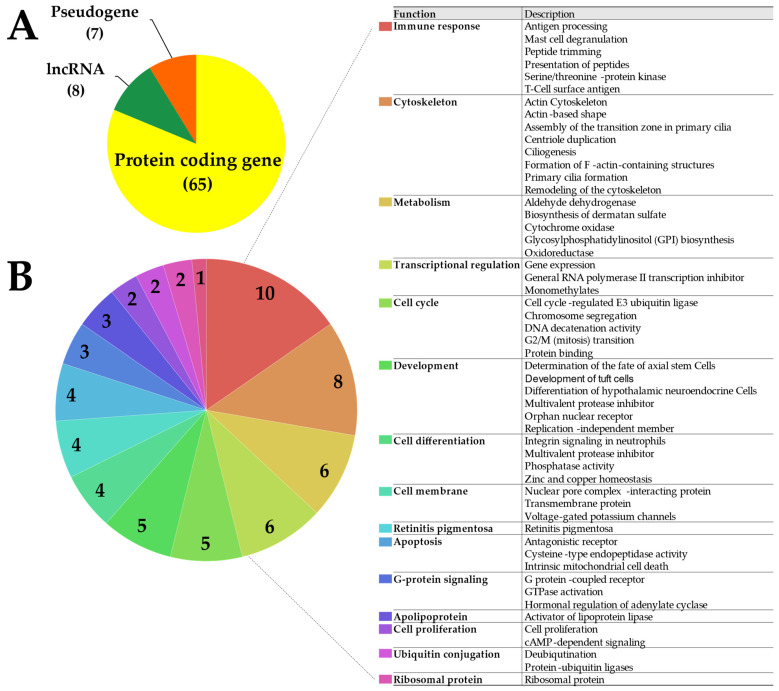
Distributions of eGenes classified by (**A**) biotypes and (**B**) functions of protein-coding genes. The numbers in each pie chart indicate the number of eGenes belonging to each category. Abbreviations: lncRNA, long non-coding RNA.

We selected 40 association signals for HL and 272 association signals for non-HL by filtering out duplication, low minor allele frequency that was <0.05, and Hardy–Weinberg disequilibrium (*p* < 1.00 × 10^−6^). After excluding 16 common signals for both HL and non-HL, transcriptome-wide eQTL analysis was conducted with 24 HL-specific signals to help infer HL-specific mechanisms (Figure 1). Overall, 80 eGenes were identified to be associated with 20 signals (*p* < 4.00 × 10^−5^, Table 1). The most significant eGene was *ERAP1*, which was associated with rs27524 (*p* = 3.60 × 10^−38^). Trans-regulation and cis-regulation were observed in 57 (71.25%) and 23 (28.75%) eGenes, respectively. These eGenes consisted of 65 protein-coding genes (81.25%), 8 long non-coding RNAs (10%), and 7 pseudogenes (8.75%) (Figure 2A). Overall, identified protein-coding eGenes had major roles in the immune system, metabolism, apoptosis, and cellular processes (Figure 2B). In particular, the cell cycle, cell differentiation, and cell proliferation categories were also determined to be involved in apoptosis.

### 3.2. Functions of eGenes

**Figure 3:**
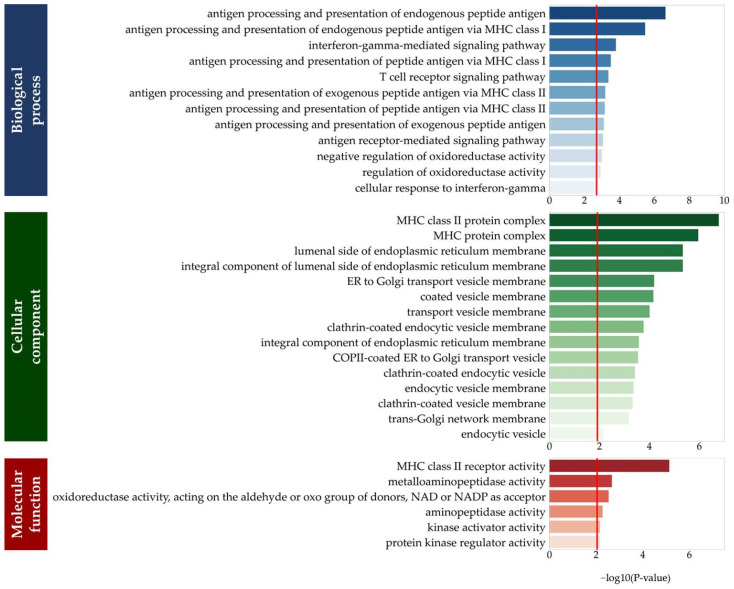
Gene Ontology (GO) term enrichment analysis for eGenes associated with HL-specific signals using Python programs from GSEApy (version 0.10.8). Red lines indicate significance, determined by multiple testing of GO terms (biological process, *p* = 1.73 × 10^−3^; cellular component, *p* = 1.23 × 10^−2^; molecular function, *p* = 9.09 × 10^−3^). Abbreviations: MHC, major histocompatibility complex; ER, endoplasmic reticulum; COPII, coat protein complex II; NAD, nicotinamide adenine dinucleotide; NADP, nicotinamide adenine dinucleotide phosphate.

Enrichment analysis revealed 174 significant functions across the 80 eGenes (*p* < 0.05, Figure 3). Multiple testing resulted in 33 functions, including 15 biological processes (*p* < 0.002), 12 cellular components (*p* < 0.012), and 6 molecular functions (*p* < 0.009).

### 3.3. Functions of Cis-Regulatory eQTL

Functional analysis of 11 cis-eQTL revealed 12 functional variants within the LD blocks of 8 eQTL in Epstein–Barr virus-transformed lymphocytes (Table 2). Seven of the 12 functional variants were identified as enhancers. In particular, rs2858870 was determined to serve as an enhancer of four genes (*HLA*-*DRB5*, *HLA*-*DQB1*, *HLA*-*DQA1*, and *TAP2*). Alternatively, rs27524 was found to be a transcriptional regulator of both *ERAP1* and *ERAP2*.

## 4. Discussion

This study identified 80 eGenes associated with HL-specific GWAS signals (*p* < 4.00 × 10^−5^), belonging to functional groups involved in abnormal cell growth and having the potential to invade other parts of the body. Specifically, these functions include cytoskeletal processes, cell cycle, apoptosis, cell differentiation, and cell proliferation. In particular, apoptosis is more critical in the pathogenesis of HL than in other cancers because apoptotic evasion by the pre-apoptotic germinal center B cells leads to malignant transformation, thereby producing RS cells, the pathological signature of HL [10,11]. Some eGenes are directly associated with apoptosis. TNFRSF10C functions as an antagonistic receptor that protects cells from apoptosis induced by TNF-related apoptosis-inducing ligand (TRAIL) activity [64]. In contrast, KHDC1 activates cysteine-type endopeptidase to induce apoptosis by cleaving caspase-8 [65]. Further, PUS10, cleaved by caspases 8 and 3 during TRAIL-induced apoptosis, may be involved in the release of cytochrome c, thereby amplifying mitochondrial apoptosis signaling [66]. Enrichment analysis using eGene sets enabled us to reveal the specific functions of these genes involved in apoptosis. In particular, terms associated with “oxidoreductase activity” were enriched in this analysis. Oxidoreductases consist of oxidase, dehydrogenase, and reductase enzymes. The current analysis resulted in the “negative regulation of oxidoreductase activity” for oxidase and “oxidoreductase activity with aldehyde/ketone as a donor and NAD/NADP as an acceptor” for aldehyde dehydrogenase (ALDH). These results are plausible considering the control of oxidoreductase: oxidase can increase reactive oxygen species (ROS) to enhance apoptosis [67], whereas ALDH can inhibit the apoptosis-inducing pathway [68]. These results aligned with a previous experimental HL study in which a decrease in ROS and a high activation of ALDH were observed (*p* < 0.05) in RS cells (L1236 and L428) [69]. Another study demonstrated a reduced expression of genes composing the NADPH complex in HL cell lines [70], suggesting that the decreased ROS in HL cells might be caused by the downregulation of NADPH oxidase. High activation of ALDH can increase glycolysis [71,72]; additionally, acetyl-CoA, required for oxidative phosphorylation (OXPHOS), has also been observed to increase in expression with enhanced glycolysis in RS cells [73,74]. Overall, the upregulated activity of OXPHOS enables the production of ATP for survival, thus evading apoptosis [74,75]. Moreover, highly activated ALDH has been reported to protect corneal cells from apoptosis by oxidizing cytotoxic 4-hydroxynonenal (4-HNE) [76].

These corresponding changes in oxidase and ALDH levels in RS cells enabled us to strongly postulate that these genes possess important roles in the pathogenesis of HL. This hypothesis may be strengthened by the eGenes identified in the present study, considering their direct regulation of oxidase and ALDH. For example, *MT3*, which is associated with the SNP rs112998813, can bind to free zinc and reduce zinc levels in cells [77,78]. Such a reduction in zinc may downregulate NADPH oxidase in a protein kinase C (PKC)-dependent manner [79]. PKC has two zinc-binding motifs and is activated in a zinc-abundant environment [80]. PKC is critical for phosphorylation-dependent assembly and activation of NOX, a key component of NADPH oxidase [81]. *OXA1L*, associated with the rs6439924 SNP, is an essential factor for translocating the N-terminal of cytochrome c oxidase subunit 2; therefore, OXA1L affects the efficiency of OXPHOS, which involves cytochrome c oxidase as complex IV [82]. Moreover, some ALDH family genes (*ALDH3A1* and *ALDH8A1*) were associated with rs7745098. ALDH3A1 can remove the presence of 4-HNE by oxidation [76]; alternatively, ALDH8A1 can oxidize a precursor to acetyl-CoA in the kynurenine pathway [83,84].

Since spurious eGenes might result from the strong linkage of the HLA genomic region, we excluded terms related to immune response (e.g., aminopeptidase and MHC) in the enrichment analysis. Nevertheless, the immune response might be important for the pathogenesis of HL since RS cells evade the immune response, thereby expanding the growth and proliferation of the RS cells within the tumor microenvironment [85].

This study showed the most significant association between *ERAP1* expression and its intronic variant rs27524 (*p* = 3.60 × 10^−38^). This strong association was supported by the GTEx study, in which the transcriptional association of *ERAP1* was found with this variant (GTEx v8, lymphocytes, *p* = 9.40 × 10^−25^ [86]; eQTLGen, whole blood, *p* = 1.78 × 10^−23^ [87]). ExPecto and Sei analyses in the current study determined that the rs27524 variant was a very functional regulator in the transcription of *ERAP1*. This nucleotide variant has also been detected in B cells via histone ChIP-seq data obtained from the Roadmap Epigenomics Consortium [61]. Moreover, the rs27524 variant may bind to 37 transcription factors that were predicted to have differential allelic binding affinity using atSNP (*p* < 0.05) [62]. In particular, NF-κB is a well-known transcription factor regulating ERAP1 expression [88] and was discovered by its motif (NFKB_disc3) in B lymphocytes (GM10847, ENCODE) [60]. Furthermore, NF-κB activity is constitutive and strong in RS cells [89,90,91]. In the GM10847 cell line, NF-κB showed a higher binding affinity to the minor (A, adenine) allele at rs27524 (*p* = 4.71 × 10^−2^) than the major (G, guanine) allele (*p* = 5.36 × 10^−1^). Moreover, BCL3, which acts as a regulator together with NF-κB in the transcription of genes, has also been discovered in B lymphocytes (K562, ENCODE) [60]. Our results indicated that BCL3 might be a repressor since it had a lower binding affinity to the A allele (*p* = 1.72 × 10^−1^) of rs27524 than the G allele (*p* = 1.80 × 10^−2^). This potential repression concurred with previous studies [92,93,94,95,96] where BCL3 bound to NF-κB to inhibit the corresponding function of the NF-κB pathway.

**Figure 4:**
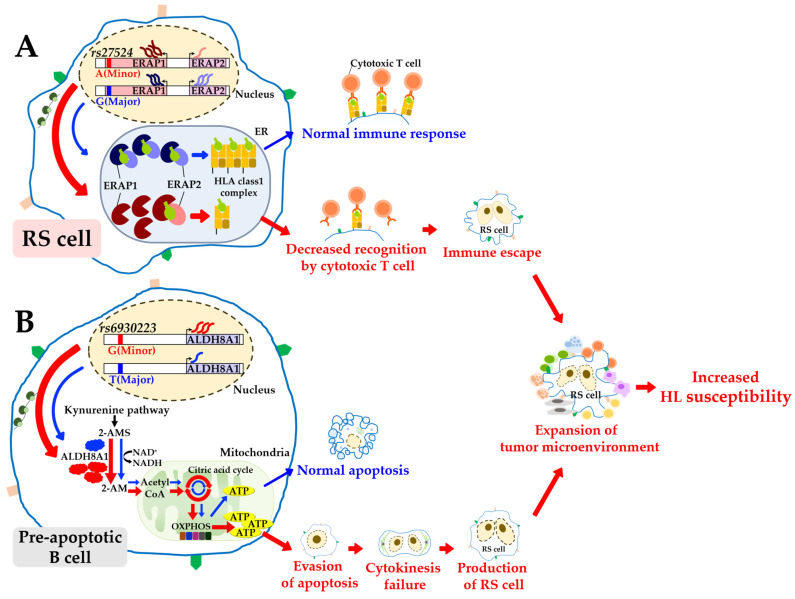
An illustrated hypothesis for the underlying mechanisms of eQTL on susceptibility to HL. Susceptibility may increase by (**A**) an imbalance between ERAP1 and ERAP2 due to the presence of the rs27524 minor allele and (**B**) enhancing the kynurenine pathway by increasing expression of ALDH8A1 due to the presence of the rs6930223 minor allele. Red and blue arrows indicate the influence of the minor (risk) and major alleles, respectively. Abbreviations: RS cell, Reed–Sternberg cell; HLA, human leukocyte antigen; 2-AMS, 2-aminomuconate semialdehyde; 2-AM, 2-aminomuconate; OXPHOS, oxidative phosphorylation.

Overall, these findings suggested that individuals homozygous for the minor allele A of rs27524 were more susceptible to HL than those homozygous for the major allele G (Figure 4A). The A allele may decrease the repressor activity of BCL3, thus inducing stronger binding of this protein to NF-κB. As a result, this NF-κB repression increases the transcriptional activity of *ERAP1*, as shown in the current study, in which *ERAP1* mRNA was observed at a higher level in those with the A allele than those with the G allele. The high ERAP1 expression with the A allele was also observed with 97 healthy individuals in a previous study [97]. On the other hand, we found that the A allele decreased the mRNA of another eGene, *ERAP2* (*p* = 1.52 × 10^−7^, Table 1). This decrease in *ERAP2* transcription may produce an undesirable imbalance between the two endoplasmic reticulum aminopeptidases, ERAP1 and ERAP2, resulting in a reduction in the supply of tumor-associated peptide antigens to HLA class 1 [98]. The attenuated HLA class 1 activity may decrease antigen presentation in RS cells and hinder the recognition of these RS cells by cytotoxic T cells [99,100]. Therefore, functional weakness of HLA class 1 may lead to immune escape and development of the tumor microenvironment in HL. Overall, this hypothesis suggests an underlying mechanism for the association signal of rs27524 which was identified from a European-based GWAS with 1200 HL cases and 6417 controls (GCST001387 [32]). Moreover, this study may explain the imbalance between ERAP1 and ERAP2 observed in various tumor cell lines, such as leukemia, lymphoma, carcinoma, and melanoma [101], and in tumor-transformed tissues of the breast, ovary, lung, colon, and thyroid [102]. The immune response escape of tumor cells by the imbalance of ERAP2/ERAP1 was further supported by prior studies [101,102], in which a decreased HLA class 1 surface expression was observed together with this imbalance, thereby reducing the tumor-associated peptide antigen presentation.

We propose another hypothesis corresponding to the second strongest eQTL signal (*p* = 9.36 × 10^−19^) of rs7745098 associated with *ALDH8A1*: individuals homozygous for its minor allele C were more susceptible to HL than those homozygous for its major allele T (Figure 4B). This eQTL was also found in previous studies (GTEx v8, lymphocytes, *p* = 5.20 × 10^−9^ [86]; eQTLGen, whole blood, *p* = 2.12 × 10^−250^ [87]). In the current study, it was determined that these associations may be attributed to a functional variant, rs6930223, in linkage with rs7745098 (LD = 0.85) because rs6930223 was detected as an enhancer variant in the transcription of *ALDH8A1* using deep learning tools (ExPecto [57]; Sei [58]), enhancer databases (Genehancer, GH06J135100 [103]; Ensembl, ENSR00001116792 [52]), histone ChIP-seq data (Roadmap Epigenomics Consortium, H3K4me1 and H3K27ac [61]), and chromatin states in B cells from the Roadmap Epigenomics Consortium (Core 15-state model [59]; 25-state model with 12 imputed marks [61]). In particular, using atSNP [62], activator protein 1 (AP1), which controls apoptosis [104] and is constitutively expressed in RS cell lines [105], was predicted, by its motif (AP1_disc10), to bind to rs6930223 in B lymphocytes (ENCODE, GM12878 [60]). Differential binding affinity was significantly observed between its alleles, G (*p* = 1.32 × 10^−5^) and T (*p* = 1.98 × 10^−1^). This suggests that the stronger binding of the enhancer variant (G allele) may increase *ALDH8A1* transcription. Increased ALDH8A1 expression enhances the oxidation of 2-aminomuconate semialdehyde to 2-aminomuconic acid, the precursor of acetyl-CoA in the kynurenine pathway [81,82]. Subsequently, acetyl-CoA upregulates OXPHOS, producing more ATP for improved survival [74,75]. As a result, this process helps these cells to evade apoptosis, thus producing RS cells 75,76]. This hypothesis explains the underlying mechanism of the HL GWAS signal rs7745098, identified from a European-based meta-GWAS with 1465 HL cases and 6417 controls (GCST002237 [33]).

In addition to these findings, we suspected that the enhanced RS cells also had weakened cytoskeletal function [106]. The current eQTL analysis revealed cytoskeleton-related eGenes (Figure 2B) with cytokinetic functions during cell division. For example, the corresponding protein of *CDC42EP5*, identified as an eGene of rs2069757, activates SEPT9, a septin protein critical for actomyosin contractility and abscission of the cell during late cytokinesis [107]. *CEP76*, an eGene of rs3806624, inhibits centriole duplication by repressing CP110 and inducing cell division failure via multipolar spindles [108]. Therefore, the failure of cytokinesis in the cell division process may be attributed to these genes, resulting in the production of RS cells with multiple nuclei [106].

## 5. Conclusions

In this study, we identified 80 eGenes from 20 HL GWAS signals. These eGenes were enriched in functions critical to HL susceptibilities, such as apoptosis, immune response, and cytoskeletal function. In particular, ERAP1 trims peptides to load onto HLA for immune responses; the minor allele at its eQTL, rs27524, may help RS cells to escape this immune response. Further, ALDH8A1 oxidizes the acetyl-CoA precursor to produce ATP for cell survival; the minor allele at rs6930223 may upregulate oxidization in pre-apoptotic germinal center B cells, resulting in apoptosis evasion. The roles of these eGenes may reflect RS cell-associated pathogenesis, a signature characteristic unique to HL. The variants, rs27524 and rs6930223, are both common over various populations, with minor allele frequency ranging from 0.27 (Latin American) to 0.43 (African) and from 0.27 (African) to 0.50 (European), respectively [109]. These minor alleles have been proposed as genetic risk factors for susceptibility to HL. Odds ratio estimates have been reported in previous European GWAS for HL; 1.22 (95% CI = 1.11–1.33) for rs27524 [32] and 1.21 (95% CI = 1.14–1.29) for rs7745098 in a strong linkage with rs6930223 (r^2^ = 0.85) [33]. Accurate risk estimates of the functional variants should be specifically obtained for different ethnic populations and/or considering different environmental exposures. Further research on the various genetic risk factors is needed to understand the genetic architecture and to improve the accuracy of precision oncology. Although this study is based on GWAS signals, the pathological scenarios were inferred largely through gene expression associated with genetic variation in normal individuals. Experimental studies on genetic risk factors for HL susceptibility, especially in patients or animal models, would elucidate the scenarios on the underlying pathogenic mechanism suggested in this study.

## Figures and Tables

**Table 1:** Identification of expression genes (eGenes) associated with HL-specific association signals ^1^.

SNP	SNP Position ^2^	Allele ^3^	MAF ^4^	eGene (chr ^5^)	Beta ^6^	*P* ^7^
rs2476601	1:114377568	G/**A**	0.10	*OTUB2* (14)	0.09	4.07 × 10^−6^
rs13034020	2:61043834	A/**G**	0.18	*PUS10* (2)	0.28	1.01 × 10^−6^
rs1432295	2:61066666	A/**G**	0.42	*PRKACB* (1)	2.55	3.44 × 10^−6^
				*RPL18A* (19)	−13.41	3.36 × 10^−5^
rs3806624	3:27764623	A/**G**	0.46	*OIP5* (15)	0.64	9.73 × 10^−6^
				*CEP76* (18)	0.11	1.23 × 10^−5^
				*H2AFZ* (4)	8.92	1.26 × 10^−5^
				*CCNB2* (15)	1.72	3.73 × 10^−5^
rs6439924	3:140169657	A/C ^U^	0.15	*RP11*-*392P7*.1 (12)	8.15	9.50 × 10^−6^
				*OXA1L* (14)	7.41	1.17 × 10^−5^
				*LA16c*-306E5.1 (16)	0.04	1.48 × 10^−5^
				*SETD6* (16)	1.18	1.57 × 10^−5^
				*ZNF311* (6)	0.03	2.36 × 10^−5^
				*RPL23AP65* (11)	26.59	2.86 × 10^−5^
				*OVCH1*-AS1 (12)	0.27	2.88 × 10^−5^
				*RPL3P9* (8)	1.38	3.11 × 10^−5^
rs20541	5:131995964	G/**A**	0.23	*NR6A1* (9)	0.42	2.02 × 10^−6^
				*PDLIM4* (5)	0.72	2.35 × 10^−5^
rs2069757	5:131998413	G/**A**	0.08	*GPRC5C* (17)	2.11	1.47 × 10^−7^
				*FBXO27* (19)	0.83	1.47 × 10^−7^
				*APOC2* (19)	1.26	3.61 × 10^−7^
				*SLC35G2* (3)	0.37	3.68 × 10^−7^
				*CD2* (1)	0.82	4.03 × 10^−7^
				*KCNRG* (13)	0.40	1.12 × 10^−6^
				*APOC4*-*APOC2* (19)	0.10	1.31 × 10^−6^
				*OTP* (5)	0.48	1.41 × 10^−6^
				*CDC42EP5* (19)	0.73	3.84 × 10^−6^
				*ZNF853* (7)	0.06	3.99 × 10^−6^
				*TMEM132B* (12)	0.30	4.05 × 10^−6^
				*DUSP15* (20)	0.84	6.44 × 10^−6^
				*SYNPO2L* (10)	0.64	6.72 × 10^−6^
				*PROSER2* (10)	0.29	8.48 × 10^−6^
				*TNFRSF10C* (8)	0.74	1.44 × 10^−5^
				*PRAM1* (19)	0.03	2.41 × 10^−5^
				*WFIKKN1* (16)	0.05	3.12 × 10^−5^
				*C12orf4* (12)	2.73	3.93 × 10^−5^
rs27524	5:96101944	G/**A**	0.35	*ERAP1* (5)	8.07	3.60 × 10^−38^
				*ERAP2* (5)	−4.84	1.52 × 10^−7^
				*EIF2AK2* (2)	1.77	1.08 × 10^−5^
rs1002658	6:137981584	C/**T**	0.16	*MTCYBP18* (5)	1.16	2.00 × 10^−5^
rs2858870	6:32572251	T/**C**	0.12	*HLA*-*DQB1*-*AS1* (6)	−4.01	7.29 × 10^−15^
				*HLA*-*DQB1* (6)	−147.20	9.36 × 10^−14^
				*HLA*-*DRB1* (6)	−118.50	5.63 × 10^−13^
				*HLA*-*DQA1* (6)	−112.40	1.39 × 10^−10^
				*HLA*-*DRB5* (6)	−54.28	5.95 × 10^−9^
				*DPYSL3* (5)	0.17	1.52 × 10^−7^
				*AC007163*.6 (2)	0.02	1.66 × 10^−5^
				*DSE* (6)	13.96	1.66 × 10^−5^
				*CHORDC2P* (14)	0.01	3.28 × 10^−5^
				*TAP2* (6)	3.38	3.76 × 10^−5^
rs649775	6:33684313	G/**A**	0.07	*RP11*-*131H24*.4 (14)	0.06	3.57 × 10^−5^
rs6928977	6:135626348	**G**/T	0.42	*AHI1* (6)	−0.75	1.44 × 10^−15^
rs7745098	6:135415004	T/**C**	0.47	*ALDH8A1* (6)	0.08	9.36 × 10^−19^
				*CTA*-*212D2*.2 (6)	0.06	5.26 × 10^−16^
				*PIGL* (17)	−0.44	9.53 × 10^−7^
				*ADNP2* (18)	−0.39	1.04 × 10^−5^
				*ALDH3A1* (17)	−0.03	1.94 × 10^−5^
				*NDUFB2*-*AS1* (7)	0.04	3.29 × 10^−5^
rs9482849	6:128288536	T/**C**	0.16	*CEP162* (6)	0.43	3.08 × 10^−5^
				*GNAI2* (3)	6.56	3.77 × 10^−5^
rs2608053	8:129075832	**C**/T	0.46	*LINC00621* (13)	0.23	3.61 × 10^−5^
rs3781093	10:8101927	T/**C**	0.17	*HSD11B1L* (19)	0.13	3.91 × 10^−5^
rs7111520	11:111249611	**A**/G	0.31	*COLCA2* (11)	−0.12	1.51 × 10^−6^
				*COLCA1* (11)	−0.06	1.78 × 10^−6^
				*CCDC13* (3)	0.20	3.16 × 10^−6^
				*HEXIM2* (17)	0.19	3.32 × 10^−6^
				*KHDC1* (6)	0.20	1.61 × 10^−5^
rs112998813	13:115059729	T/**C**	0.08	*UPF3AP2* (17)	−1.56	8.10 × 10^−10^
				*CDC16* (13)	2.94	1.24 × 10^−8^
				*MT3* (16)	1.06	6.78 × 10^−6^
				*AL928768*.3 (14)	112.90	6.95 × 10^−6^
				*ZNF534* (19)	0.06	7.98 × 10^−6^
				*TATDN1* (8)	0.72	8.03 × 10^−6^
rs6565176	16:30174926	C/**T**	0.43	*RP11*-345J4.5 (16)	−2.52	2.43 × 10^−10^
				*TBX6* (16)	0.07	3.09 × 10^−8^
				*RP11*-*166B2*.1 (16)	0.55	8.92 × 10^−7^
				*AC006014*.7 (7)	−0.16	1.29 × 10^−5^
				*NPIPB11* (16)	0.61	2.15 × 10^−5^
				*FAM13B* (5)	0.44	3.38 × 10^−5^
rs1860661	19:1650134	**A**/G	0.40	*RP11*-*93B14*.6 (20)	0.01	1.29 × 10^−5^

^1^ Only significant eGenes with *p* < 4.00 × 10^−5^; ^2^ Formatted as “chromosome number:base pair (bp)” from GRCh37; ^3^ Major/minor allele. Bold nucleotide indicates risk allele and superscript U indicates unknown risk allele; ^4^ Minor allele frequency; ^5^ Chromosome number of eGene from GRCh37; ^6^ Regression coefficient estimated using a mixed model under the assumption of an additive genetic effect. Positive value of the regression coefficient estimate means that the minor allele of the SNP increased expression of the eGene, and negative value means that the minor allele decreased expression of the eGene; ^7^ Probability of obtaining the regression coefficient more extreme than the estimate under the assumption that regression coefficient is zero.

**Table 2:** Functional single nucleotide polymorphisms (fSNPs) linked to the SNPs that were identified as eQTL (iSNPs) ^1^.

iSNP	eGene	fSNP			LD ^4^	Regulatory Function ^5^
		ID	Position ^2^	Allele ^3^		
rs13034020	*PUS10*	rs1432296	2:61068167	C/**T**	0.82	Promoter
rs27524	*ERAP1*	rs27524	5:96101944	**G**/A		Transcription
	*ERAP2*	rs27524	5:96101944	**G**/A		Transcription
rs6928977	*AHI1*	rs2746432	6:135696597	T/**C**	0.86	Transcription
rs2858870	*HLA*-*DRB5*	rs17840121	6:32577693	G/**C**	0.87	Enhancer (multi-tissue)
	*HLA*-*DQB1*	rs28383358	6:32606779	**G**/A	0.84	Enhancer (B-cell-like)
	*HLA*-*DQA1*	rs28383344	6:32605067	C/**G**	0.93	Enhancer (multi-tissue)
	*HLA*-*DRB1*	rs28366261	6:32559572	G/**C**	0.85	Transcription factor CEBPB
	*TAP2*	rs115110712	6:32600340	A/**G**	0.97	Enhancer (brain)
rs7745098	*ALDH8A1*	rs6930223	6:135424203	T/**G**	0.85	Enhancer (B-cell-like)
rs7111520	*COLCA2*	rs4283016	11:111248640	**G**/A	0.81	Enhancer (B-cell-like)
rs112998813	*CDC16*	rs17337612	13:114998977	**G**/C	0.87	Enhancer (erythroblast-like)
rs6565176	*TBX6*	rs9924308	16:30154740	G/**A**	0.97	CTCF-Cohesin

^1^ fSNP was discovered with its regulatory functions to the corresponding eGene using ExPecto framework [57]; ^2^ Formatted as “Chromosome number:bp” from GRCh37; ^3^ Major/minor allele. Bold nucleotide indicates a larger regulatory effect on the eGene; ^4^ Linkage disequilibrium (r^2^); ^5^ Regulatory functions were predicted using deep learning sequence models in the Sei framework. The parentheses for “regulatory function” indicate the tissues in which the fSNP was reported to regulate [58].

## Data Availability

The data used in this study are publicly available in the GEO DataSets (Accession No. GSE61742).
